# A novel workflow for indirect cobalt-chromium restorations using additive manufacturing without digital design

**DOI:** 10.34172/joddd.2021.025

**Published:** 2021-08-25

**Authors:** Les Kalman, Lyndsay Desimone

**Affiliations:** ^1^Department of Restorative Dentistry, Schulich School of Medicine & Dentistry, Western University, London, Ontario, Canada; ^2^Graduate Student, Western University, London, Ontario, Canada

**Keywords:** 3D printing, Chromium-cobalt alloy, Restorations, Workflow

## Abstract

This preliminary investigation explored additive manufacturing to fabricate cobalt-chromium onlay restorations without the use of digital design. Extracted molars were prepared for four-surface onlays followed by the conventional approach for the fabrication of provisionals. The provisionals were digitized with an intraoral scanner, and stereolithography (STL) files were fabricated with additive manufacturing in cobalt-chromium, utilizing selective laser melting (SLM). Onlays were bonded to the corresponding tooth. Restorations were polished after cementation and assessed with photography, radiography, and a clinical post-cementation checklist. Cementation was unremarkable; marginal adaption and surface finish were generally acceptable. A simple, efficient, and inexpensive alternative workflow for the fabrication of indirect restorations without using the digital design is proposed.

## Introduction

The impact of technology on dentistry has substantially increased over the last decade.^1^ This has been well documented in prosthodontics with the increased use of digital impressions and digital design for indirect restorations.^2,3^ Although the technology has significantly changed, the workflow has not. Procedural steps include tooth preparation, digitization, digital design, and restoration fabrication, respectively.^3^ The established workflow can prove challenging in terms of time, cost, and complexity, limiting its application and availability.^4^

This preliminary in vitro investigation explored the use of additive manufacturing to fabricate cobalt-chromium onlay restorations without a digital design.

## Methods

**Figure 1:**
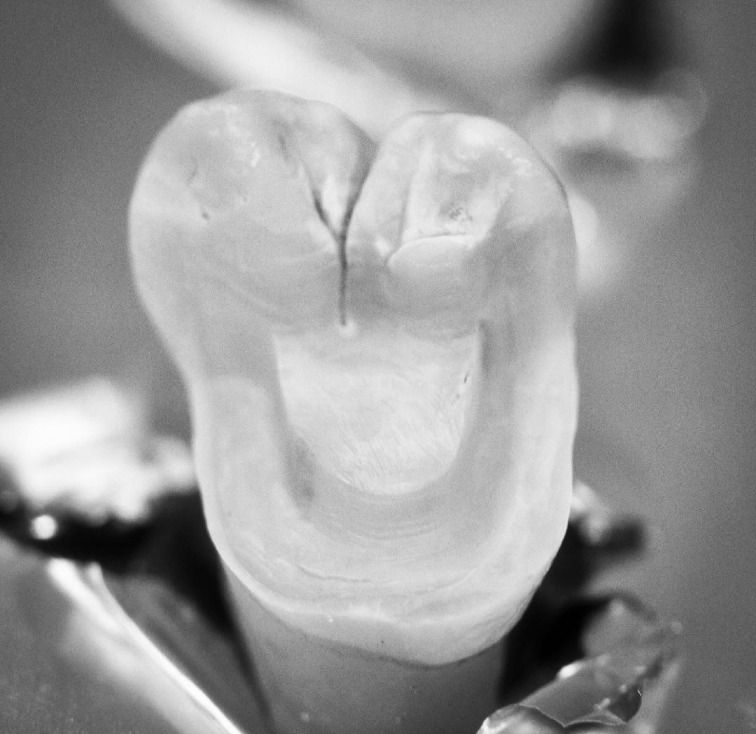
Tooth preparation (MODL).

**Figure 2:**
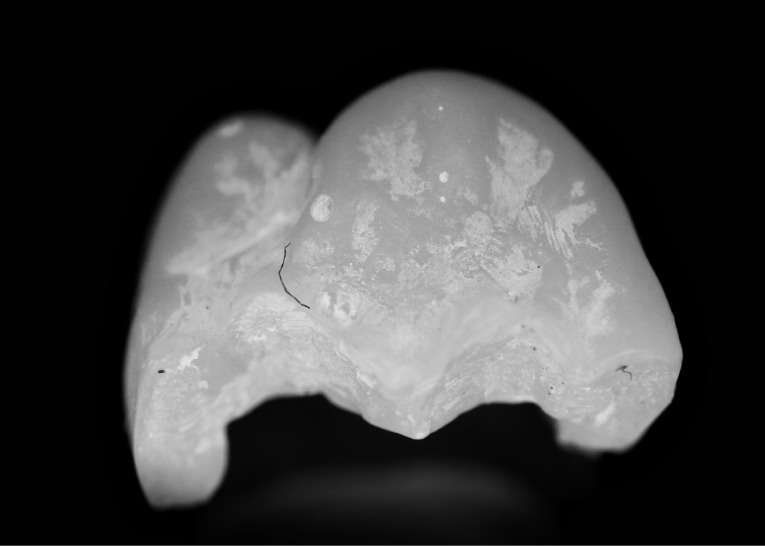
Provisional onlay restoration.

**Figure 3:**
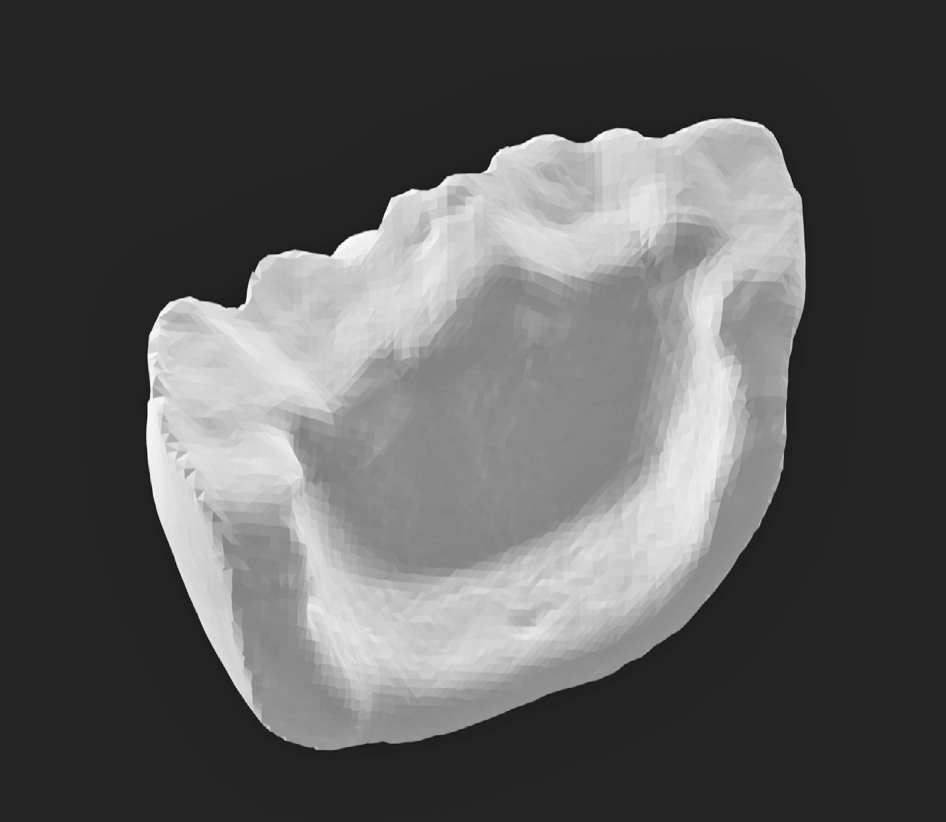
Intaglio view of the STL file of the onlay.

**Figure 4:**
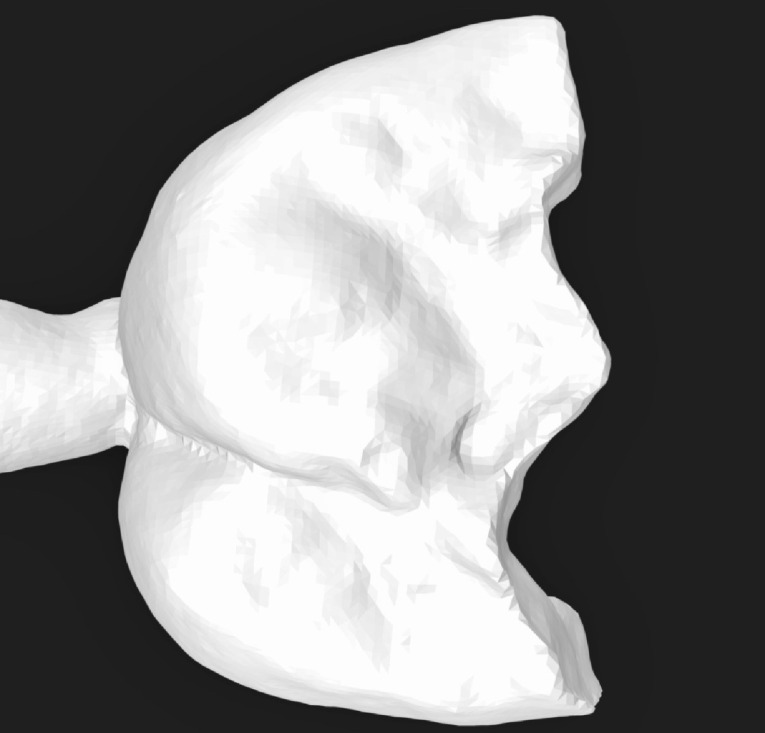
Occlusal view of the STL file of the onlay.

**Figure 5:**
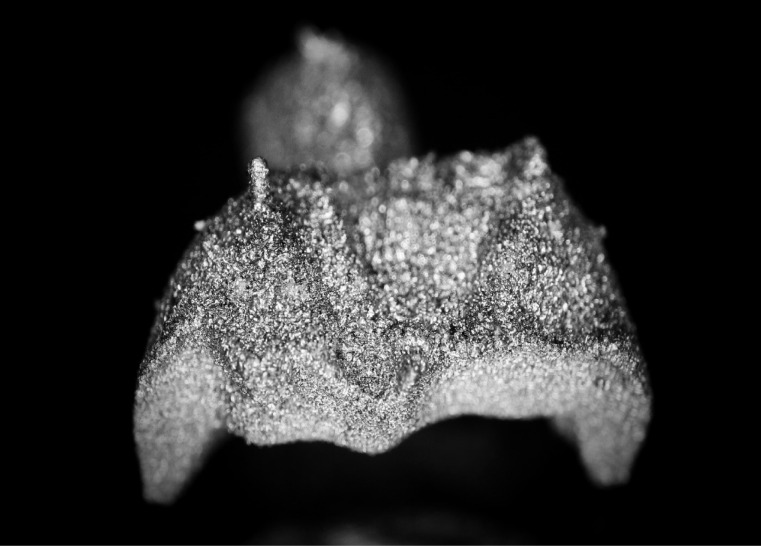
Occlusal view of the additive-manufactured onlay.

**Figure 6:**
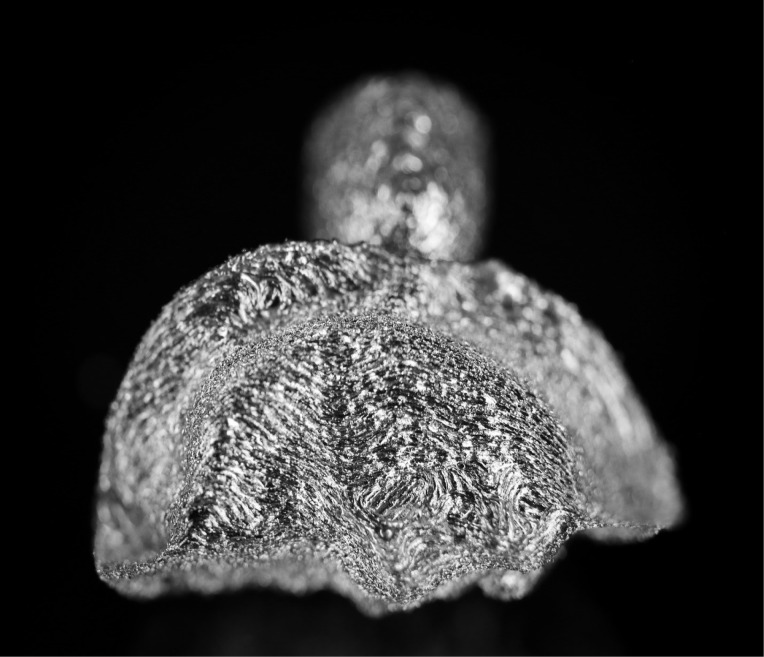
Intaglio view of the additive-manufactured onlay.

Three extracted, undamaged human molar teeth were randomly selected from a group of extracted molars. Each tooth was fixed onto a hand-held support. A sectional tray (TempTray; Clinician’s Choice, London, Canada) and provisional material (Template; Clinician’s Choice, London, Canada) were employed to impress each tooth. The teeth were prepared for a four-surface lithium disilicate (Ivoclar, Buffalo, United States) onlay preparation (Figure 1), as either a mesial-occlusal-distal-lingual (MODL) or a mesial-occlusal-distal-buccal (MODB) onlay. The provisional matrix was filled with a bis-acrylic composite resin (Integrity; Dentsply, Milford, United States) and placed over the prepared tooth. A standard four-surface provisional restoration was then fabricated, shaped, and polished (Figure 2). The provisional restorations were fixed with a Pic-n-Stic (Pulpdent, Watertown, United States) and coated with titanium dioxide (3M ESPE, St. Paul, United States). The provisional restorations were then digitized (True Definition; 3M ESPE, St. Paul, United States) to create a digital stereolithography (STL) file of the provisional restoration (Figures 3 and 4). The STL files were digitally transferred to ADEISS (London, Ontario). The files were imported with Fusion 360 software (Autodesk, San Rafael, United States) and printed in cobalt-chromium (Figures 5 and 6) with a Renishaw AM 400 Laser Melting System (Renishaw PLS, Gloucestershire, United Kingdom). The printer utilized selective laser melting (SLM) technology, which melted and fused metallic powder layers (with an average diameter of 30‒50 µm) using a 400-W laser. The onlay restorations underwent standard post-processing without polishing.

**Figure 7:**
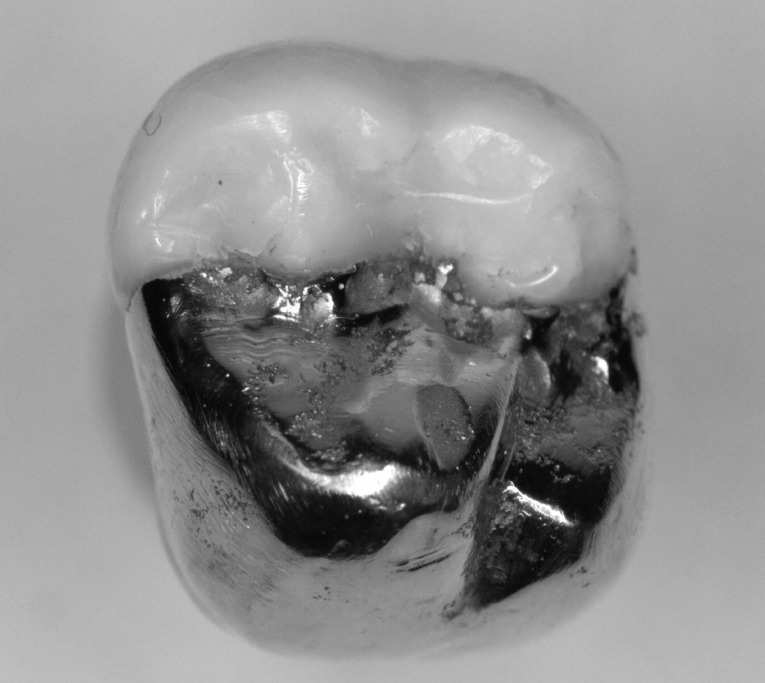
Photograph of the occlusal view of cemented onlay.

**Figure 8:**
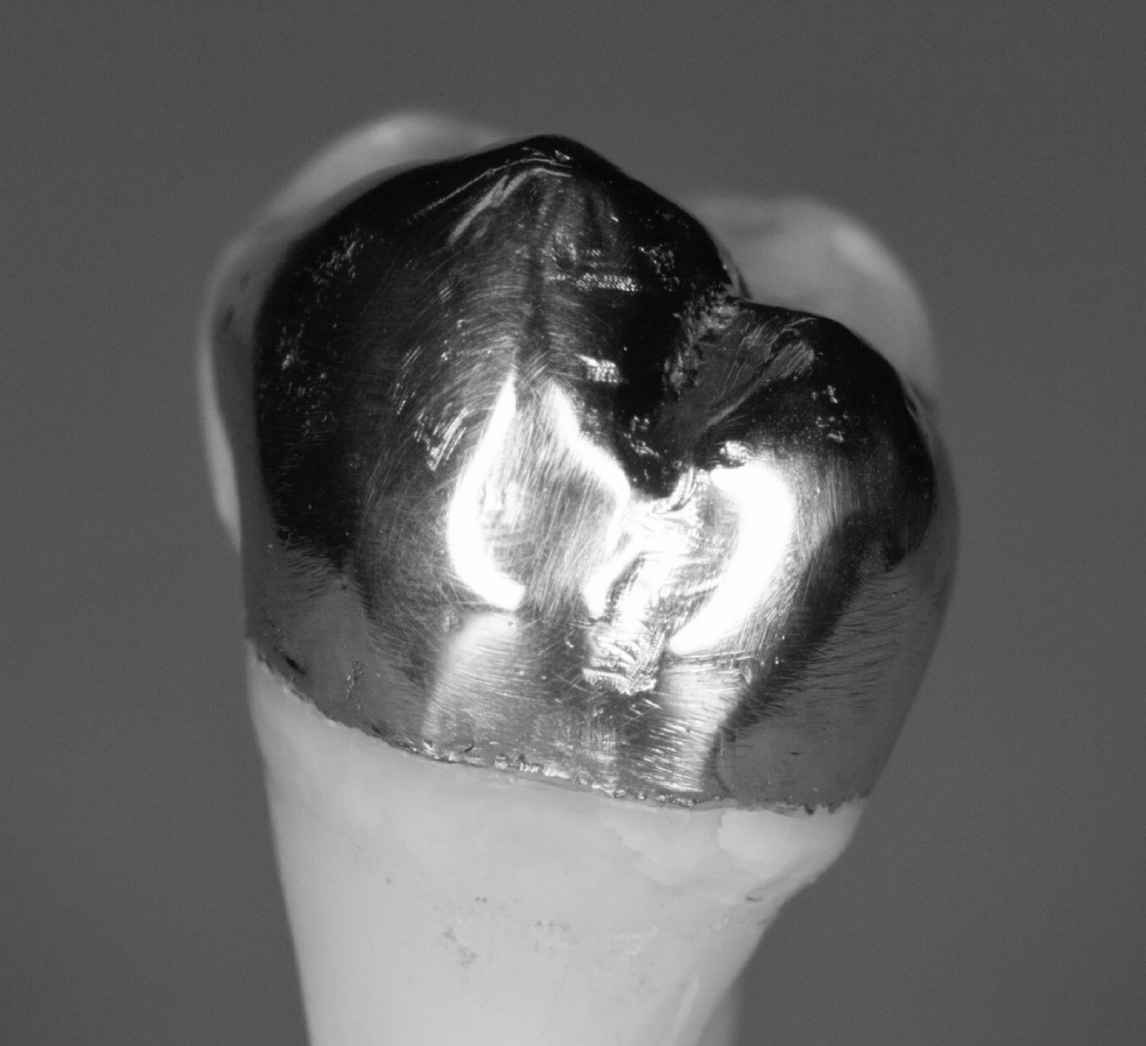
Photograph of the palatal view of cemented onlay.

**Figure 9:**
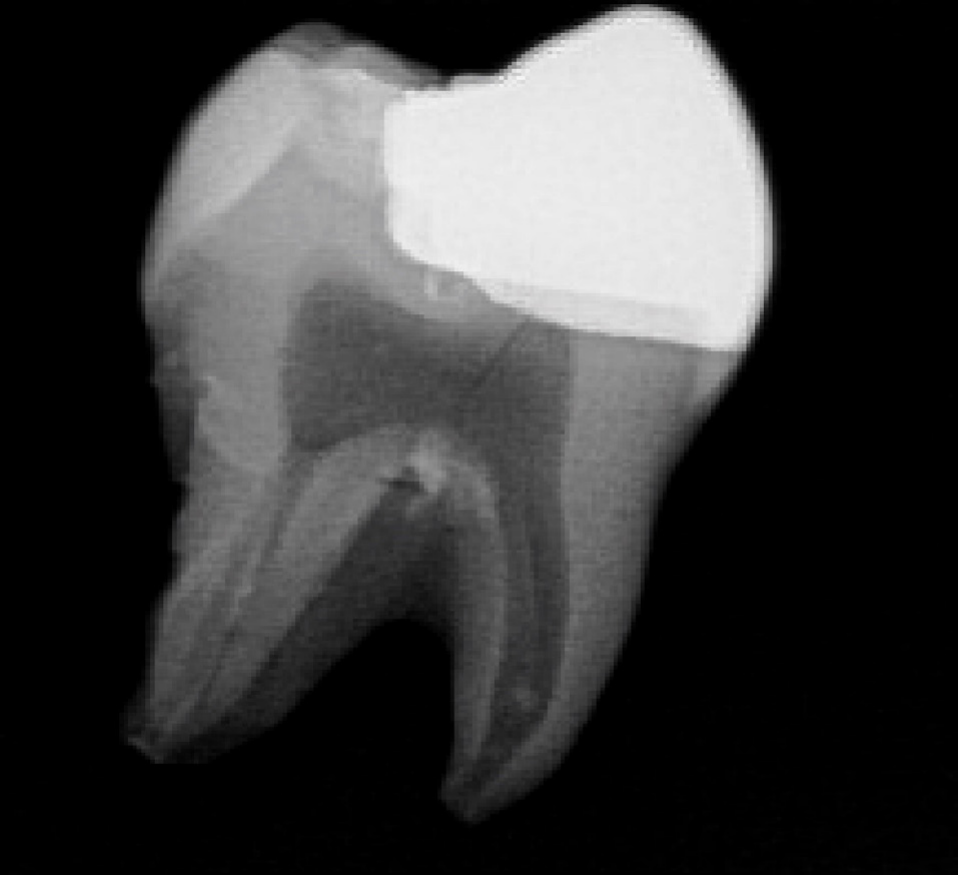
Mesial-distal radiographic view of the cemented onlay.

**Figure 10:**
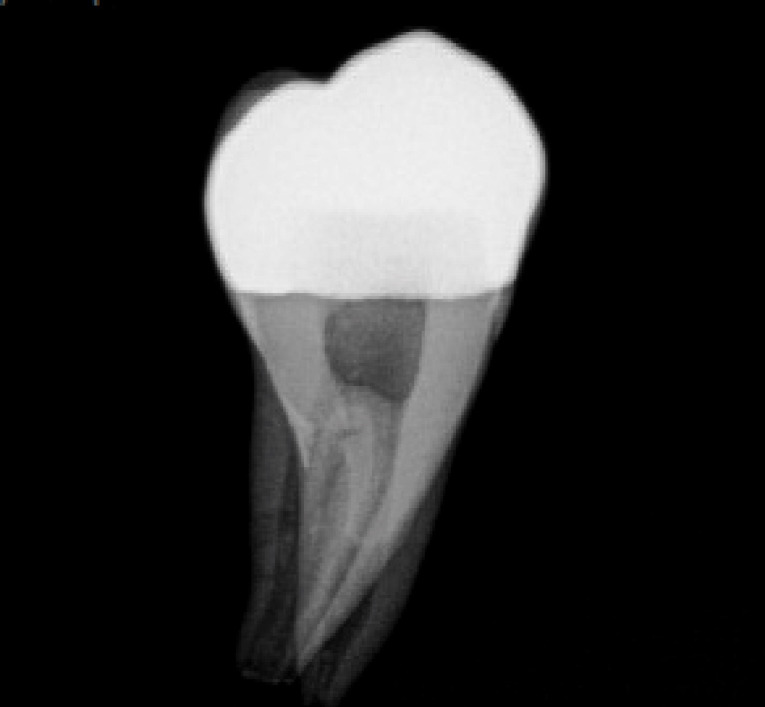
Buccal-lingual radiographic view of the cemented onlay.

Each onlay was bonded to its corresponding prepared tooth using resin cement (GC, Alsip, United States) following the manufacturer’s protocol.^5^ The restorations were then polished with high-speed diamond burs and slow-speed finishing discs. Post-cementation photographs were taken of the restorations in buccal, lingual, mesial, distal and occlusal views (Figures 7 and 8). Radiographs were also taken for each restoration in mesiodistal and buccolingual aspects (Figures 9 and 10).

## Results

The digital scanner provided an STL file of suitable resolution for three-dimensional (3D) printing in metal. The indirect onlay restorations were successfully 3D-printed in cobalt-chromium with dimensions, morphology, and fit clinically acceptable for subsequent cementation. Cementation was unremarkable and adequately retained the onlays, similar to previous investigations.^6^ Marginal adaption was generally acceptable but was clinically unacceptable in one area due to an open margin. The surface finish was generally acceptable but could be improved in some areas, especially on the occlusal surface.

## Discussion

This preliminary study demonstrated that the workflow of additive manufacturing of cobalt-chromium onlays, without digital design, is possible. For predictability, the scanner and printer must have an adequate resolution. The printed final restoration quality depends on the provisional; therefore, a highly morphologically accurate, ideally adapted (margins), and polished provisional is mandatory, as cobalt-chromium is difficult to polish. The preparation was suitable for the material.

**Figure 11:**
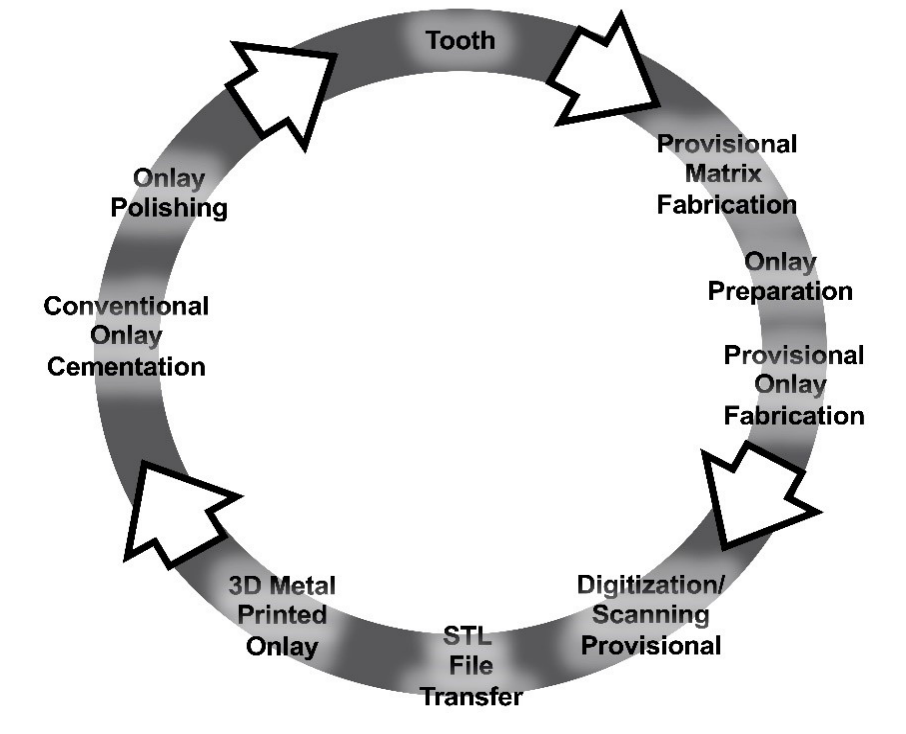
Proposed novel workflow for indirect cobalt-chromium restorations using additive manufacturing without digital design.

A novel workflow has been proposed (Figure 11) that provides the clinician with a simple, efficient, and inexpensive alternative to the traditional workflow. The novel workflow bypasses the need for digital design, which would significantly reduce (1) the time required and (2) the cost compared to the conventional workflow. This approach might be warranted where costs create barriers to treatment for both the patient and clinician.

**Figure 12:**
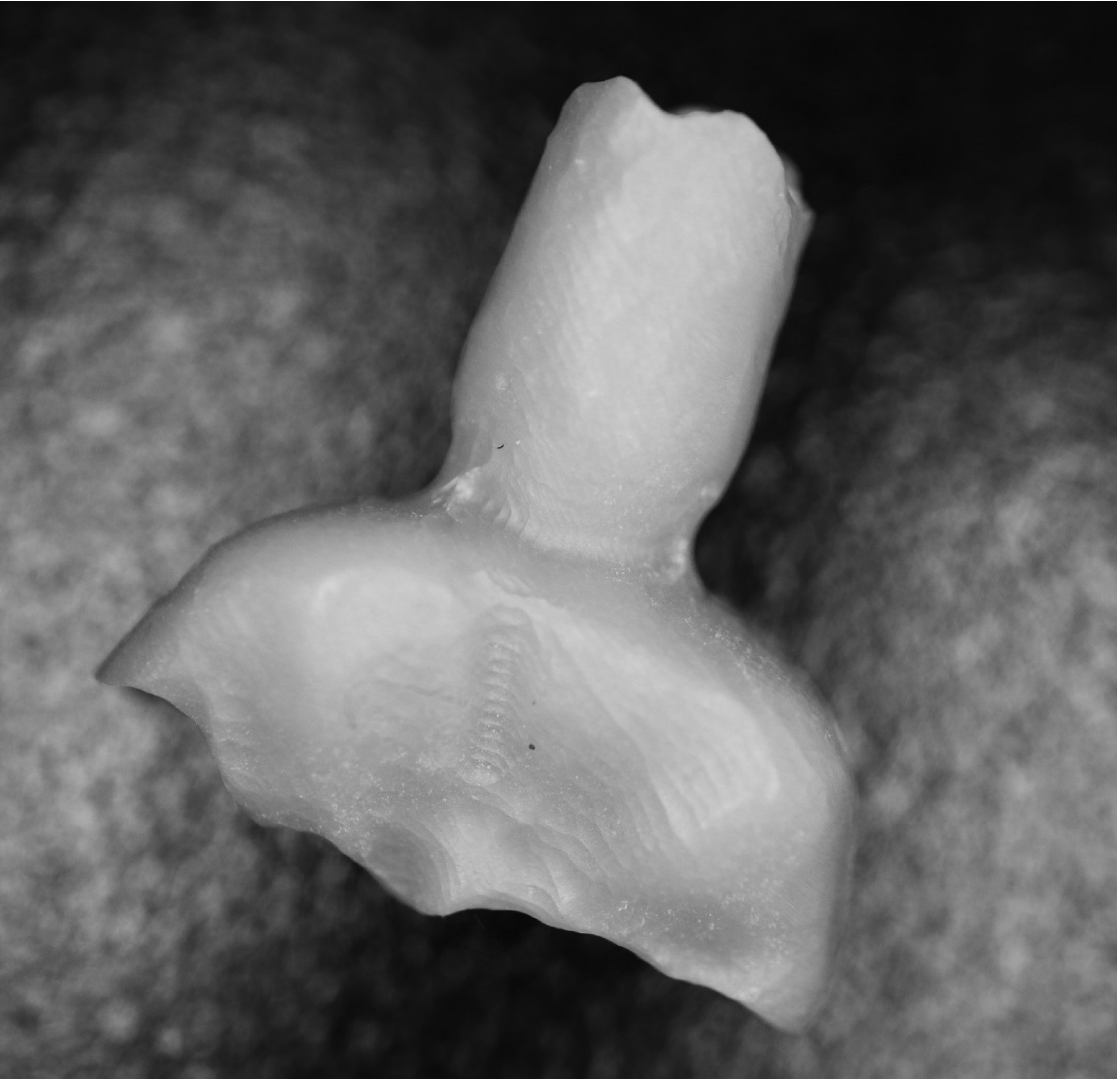
Occlusal view of the milled zirconia onlay.

**Figure 13:**
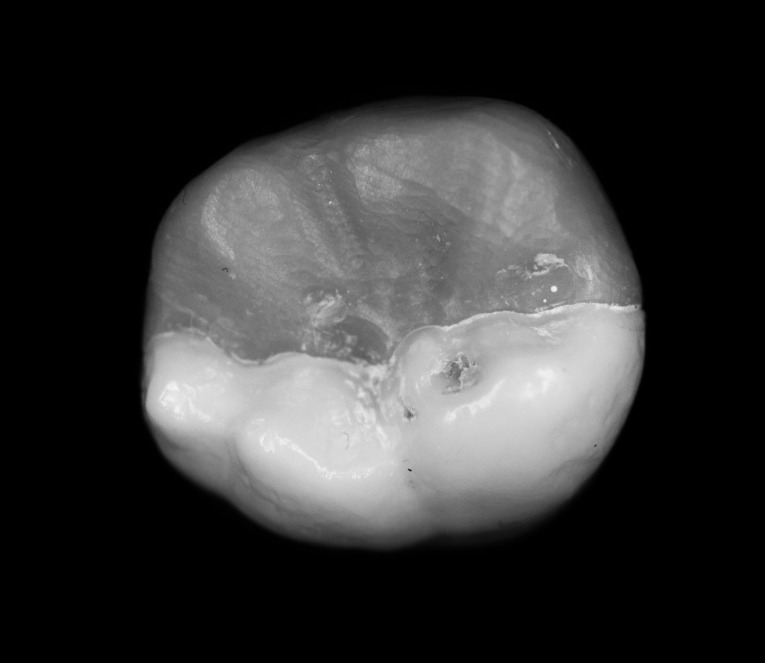
Photograph of the occlusal view of cemented zirconia onlay.

Although this study investigated indirect restorations with cobalt-chromium, other metal powders currently employed in additive manufacturing could be utilized, including stainless steel and titanium.^7^ Moreover, the novel workflow could be applied to non-metal, esthetic restorations, including zirconia and lithium disilicate, using milling (CAD/CAM) as the output. The authors have investigated this with a single unit with the same workflow and similar results (Figures 12 and 13).

This preliminary study had a small sample size, and the assessment was limited, as the restorations were only assessed through (1) photographs, (2) radiographs, and a (3) clinical post-cementation checklist on stand-alone extracted teeth. Further evaluations are required with larger sample sizes, adjacent teeth, antagonists, other materials, physical testing, and a clinical study.

## Conclusion

Digital dentistry will continue to expand and have an increased impact on clinical practice. The novel workflow for indirect cobalt-chromium restorations using additive manufacturing without digital design provides clinicians and patients with an alternative approach. The simplified workflow, with significantly reduced time and cost, provides a predictable and successful technique for indirect restorations.

## Authors’ Contributions

Conceptualization: LK. Investigation: LK. Methodology: LK. Supervision: LK. Writing the original draft: LK. Review and editing: LK & LD. All the authors have read and agreed to the published version of the manuscript.

## Acknowledgments

The authors acknowledge Dr. Vishal Patel for the onlay preparations on extracted teeth, Ashwin Baskaran and Megan Checora for assisting with the polishing, ADEISS for the 3D metal printing, and Alien Milling Technologies for the zirconia restoration.

## Funding

Western University’s Schulich Dentistry provided funding for the research through an Internal Research Grant (IRG).

## Competing Interests

The authors declare no competing interests with regards to the authorship and/or publication of this article.

## Ethics Approval

Not applicable.
